# A Novel Demography-Based Approach to Define Patient-Specific Outflow Boundary Conditions in CT-Based FFR Computations

**DOI:** 10.1007/s10439-026-04002-2

**Published:** 2026-03-03

**Authors:** Ernest W. C. Lo, Francesca Pugliese, Leon Menezes, Ryo Torii

**Affiliations:** 1 Department of Medical Physics and Biomedical Engineering, UCL EPSRC CDT for Medical Imaging, University College London, Gower Street, London, WC1E 6BT UK; 2 Barts Heart Centre, Barts Health NHS Trust, London, UK; 3 Centre for Advanced Cardiovascular Imaging, William Harvey Research Institute, Queen Mary University of London, London, UK; 4 NIHR Barts Biomedical Research Centre, London, UK; 5 UCL Institute of Nuclear Medicine, NIHR University College London Hospitals Biomedical Research Centre, 235 Euston Rd, Bloomsbury, London, NW1 2BU UK; 6 Department of Mechanical Engineering, University College London, Torrington Place, London, WC1E 7JE UK

**Keywords:** Fractional flow reserve, Computational fluid dynamics, Outflow boundary condition, Perfusion imaging

## Abstract

**Background and Objective:**

Computed tomography-based fractional flow reserve computation (CT-FFR) is widely used in clinical practice, based on its efficacy demonstrated in many studies. However, major assumptions remain with the outflow boundary conditions (BCs) representing coronary microvasculature, especially in hyperaemia. We here propose a novel method to estimate patient-specific microvascular response to hyperaemia for CT-FFR calculations, based on patients’ routinely available demographic data.

**Methods:**

A statistical model to predict microvascular flow response (MFR) from routinely collected patient demographic parameters was derived using PET-based perfusion data of 101 patients with coronary artery disease. CT-FFR computations were then conducted with patient-specific anatomical models and outflow BCs derived from various MFR models including the proposed approach. The FFR values were calculated for an independent test cohort of 10 patients who had undergone CT coronary angiography, CT perfusion imaging and invasive FFR measurement. Computed FFR values were compared against invasive FFR and other CT-FFR algorithms.

**Results:**

A multivariate regression model predicting patient-specific MFR was derived as a function of sex, diabetes and smoking status of the patient. FFR values computed using our model agreed well with the invasive FFR (0.76 ± 0.09 vs. 0.75 ± 0.10, *P* = 0.217). The FFRs predicted with our model were also comparable to those calculated using outflow BC tuned with patient-specific perfusion data (FFR: 0.74 ± 0.10, *P* = 0.233 vs. invasive FFR) and showed marked improvement over the conventional approach (FFR: 0.68 ± 0.11, *P* = 0.004 vs. invasive FFR). Diagnostic accuracy vs. invasive FFR were 100, 91 and 82% for CT-FFR with CTP-based MFR, demography-based MFR, and conventional approach, respectively.

**Discussion:**

The proposed demography-based MFR model significantly improves FFR computation accuracy compared with a typical conventional model that assumes constant, healthy and population average MFR. Although its diagnostic accuracy is slightly lower than that of CT-FFR calibrated with patient-specific perfusion imaging data (91 vs. 100%), the demography-based model offers a substantial practical advantage by not requiring additional non-standard data acquisition, such as perfusion imaging. Consequently, it shows strong potential as a practical enhancement to conventional CT-FFR algorithms.

## Introduction

Computation of fractional flow reserve (FFR) based on computed tomography (CT) images and computational fluid dynamics (CFD) has become a common diagnostic procedure. CT-based FFR computation (CT-FFR) is used as a non-invasive version of the catheter-based FFR measurement for the functional assessment of an epicardial stenosis [1], often utilised as a pre-screening step before invasive measurement [2]. By simulating blood flow within coronary vessels instead of catheterisation, CT-FFR estimates the pressure ratio across a stenosis and provides clinicians with a functional metric to support interventional planning. In conventional CT-FFR approaches, such as HeartFlow FFR_CT_ [1, 3, 4], coronary anatomy is reconstructed from CT Coronary Angiogram (CTCA) images and used in the calculation of FFR, together with patient-specific cardiac parameters including blood pressure and heart rate. The application of non-invasive FFR computation has grown with accelerated surrogate models using machine learning [5], some not even including CFD [6], and expanded to be used with other imaging modalities such as X-ray angiograms [7, 8].

One of the remaining challenges associated with CT-FFR is outflow boundary conditions including the representation of hyperaemic conditions. Developers of FFR_CT_ framework have reported “models of adenosine-mediated hyperaemia may overestimate the degree of vasodilation”, resulting in FFR_CT_ values overestimated in patients with microvascular disease [1]. Our previous study showed that outflow boundary conditions (BCs) in CT-FFR computations, reflecting the degree of hyperaemic response per patient, significantly alter the predicted FFR values especially when the stenosis is tight [9]. The study was conducted by calibrating the resistance of peripheral coronary vasculature using positron emission tomography (PET) imaging. Myocardial perfusion imaging (MPI) such as PET is currently considered the gold standard for regional ischaemia detection [10]. However, because MPI alone can detect ischaemia, MPI is not generally acquired for the patients who undergo CT or invasive FFR [11, 12]. Moreover, a key advantage of current CT-FFR algorithms is that they rely solely on CTCA images. CTCA are becoming more routinely available under the recommendations by medical bodies such as UK’s National Institute for Health and Care Excellence (NICE) as a first-line investigation for suspected coronary artery disease (CAD) patients [13]. To acquire MPI for the purpose of providing outflow BC for CT-FFR is not clinically viable as it could incur additional radiation dose to the patient, and would impose additional resources to the healthcare system. It is therefore desirable to estimate the patient-specific vasodilatory capacity of the microvasculature without further imaging.

To incorporate vasodilatory capacity, one approach is to model the multi-scale structure from the large epicardial artery to the microvascular perfusion [14]. Such multi-scale approaches are emerging since last years [15, 16], and has an advantage of providing a detailed mechanistic insight to the complex interaction between the epicardial vessels and microvasculature. At the same time, their limitation is the large computational resource and time required, as well as additional model set up and input parameters for the perfusion model. Myocardial perfusion model framework for clinical use has been proposed [17], and coupled with simplified epicardial vessel models [18], but not used for improved FFR computations. Another approach is to use a more empirical method. Knott et al. derived an empirical relationship between stress myocardial blood flow (MBF) and age, diabetes, left ventricular (LV) mass and beta-blockers, in patients with non-obstructive coronary disease using perfusion magnetic resonance imaging (MRI) [19]. This is more practical, not requiring additional computing power in CT-FFR computations and yet based on standard demographic data.

We therefore aimed to develop a novel approach to CT-FFR computation that incorporates individualised outflow BCs based on demographic and imaging data which are routinely available in clinics. More specifically, objectives were set to (1) develop a statistical model to predict individual hyperaemic response based on demographic data, (2) incorporate the model to previously-developed CT-FFR protocol [9] and (3) validate the results against invasive FFR measurements. Once validated, this novel outflow BC model would enhance the accuracy hence the utility of CT-based FFR computation, without the need of any additional data collection such as perfusion imaging which requires additional facility and time in the process.

## Methods

### Patient Datasets

In this study, two patient datasets were used: PET dataset and CT perfusion (CTP) dataset. Written informed consent was obtained from all patients, in accordance with the Declaration of Helsinki. The PET data set was obtained in accordance with the recommendations of the South East Research Ethics Research Committee (Aylesford, Kent, UK). The study protocol involving CT and invasive FFR for the CTP cohort was approved by the East Central London Research Ethics Committee (10/H0721/79).

#### PET Cohort

This dataset of 101 patients (65 male, 36 female, age 70 ± 9 years) was used to derive a statistical model of coronary flow reserve (CFR) as a function of demographic parameters. The patients presented chest pain and other symptoms that indicated an intermediate risk of CAD at UCL Hospital. All patients underwent 82Rb PET perfusion imaging using GE Discovery STE scanner to identify ischaemic regions in the myocardium. The CFR values in this dataset were calculated using Siemens Syngo as part of routine clinical diagnostic workflow. Demographic metrics such as age, sex, smoking status, diabetes, and family history were also recorded for clinical diagnostic purpose, summarised in Table 1, which were used as the basis of the statistical model to predict a patient-specific CFR.

**Table 1:** Patient demographics of the two cohorts in this study

	PET cohort		CTP cohort	
Number of patients	101		10	
Number of stenosed vessels	–		11	
Age (years)	70 ± 9		59 ± 6	
Male sex (%)	65 (64)		10 (100)	
Diabetes	22		2	
Smoking	24		8	
Hypertension	56		6	
Hyperlipidaemia	59		9	
Family History	51		2	
Pain Limitation	47			
Breathlessness	49			
Image-based global CFR	1.81 ± 0.47		2.36 ± 0.52	
Image-based MFR (global CFR without territory suspected for stenosis)	1.89 ± 0.48			
Invasive FFR	–		0.75 ± 0.097	
	Rest	Stress	Rest	Stress
Heart rate	70.3 ± 12.8	87.9 ± 16.3	65.1 ± 11.8	84.9 ± 16.3
Systolic BP	131.1 ± 22.0	126.3 ± 20.4	139.2 ± 19.0	137.8 ± 18.0
Diastolic BP	73.7 ± 12.6	69.7 ± 11.7	76.5 ± 10.5	73.1 ± 8.7

#### CTP Cohort

This dataset of 10 patients (10 male, 0 female, age 59 ± 6 years) was used as validation/test dataset. The patients underwent CTCA and CT perfusion (stress and rest) imaging at Barts Heart Centre, using Siemens SOMATOM Definition Flash, for suspected CAD. Demographic data were recorded and presented in Table 1 along with the PET dataset. All patients also underwent invasive FFR at Barts Heart Centre.

### Procedure of CT-FFR Computations

#### Anatomical Model Reconstruction and Meshing

**Fig. 1:**
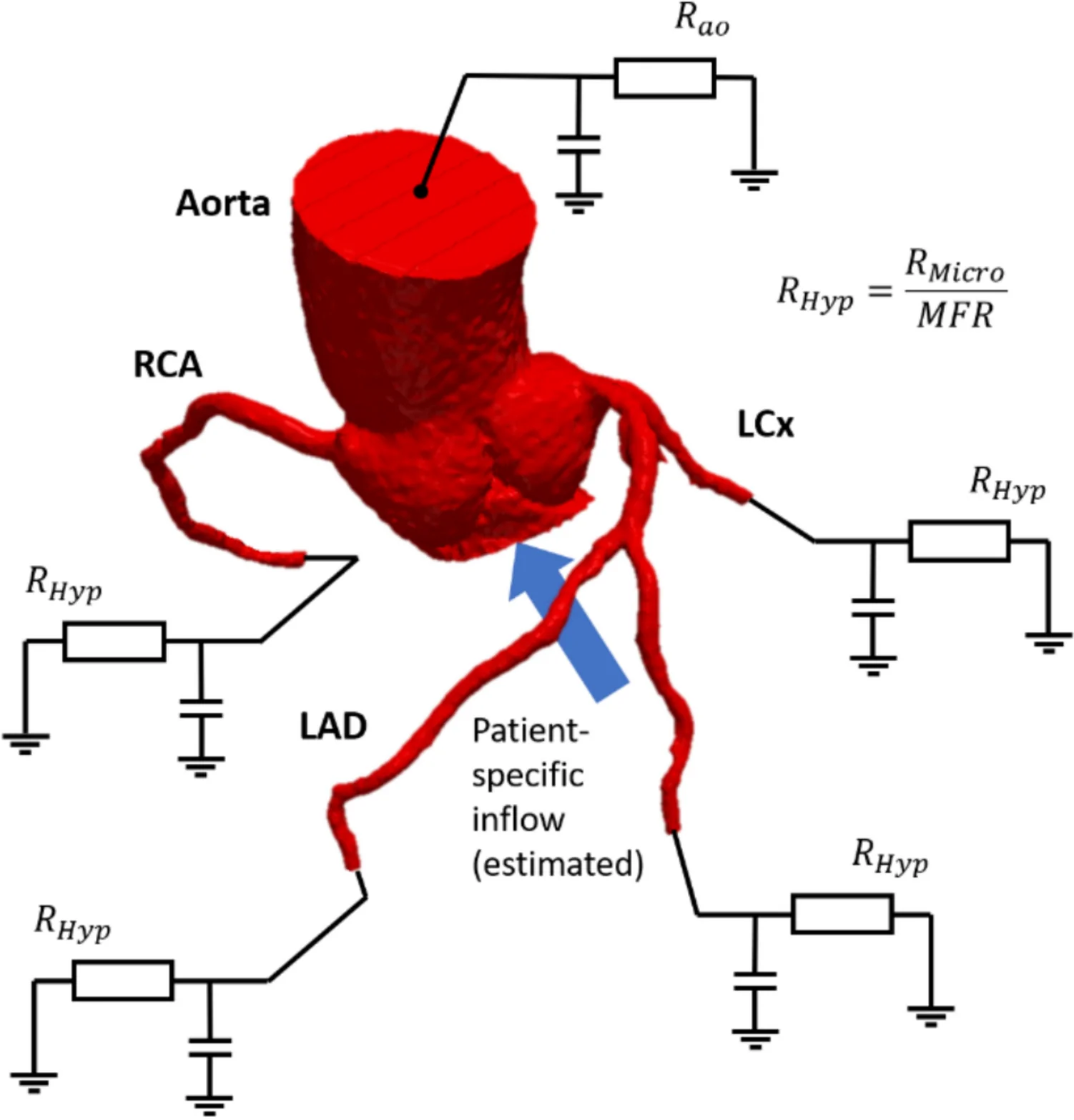
An example of anatomical model reconstructed from CTCA images, with indication of inflow and outflow boundary conditions. Note that during hyperaemia, the downstream microvascular resistance is reduced: ${R}_{Hyp}$, which is the baseline microvascular resistance ${R}_{Micro}$ divided by the Microvascular flow response MFR

CTCA images of CTP dataset were segmented to reconstruct 3D anatomical models of coronary artery tree using Simpleware ScanIP (Synopsys, CA, USA). The segmentation was conducted by region growing with a Hounsfield unit threshold of approximately 200 and no smoothing was performed. Branches of their diameter smaller than 2 mm were excluded due to the resolution limit of CTCA: 0.346 mm in-plane pixel size and 0.5 mm slice interval. An example of reconstructed coronary tree is shown in Fig. 1. Volumetric mesh for CFD was generated also using ScanIP. Tetrahedral elements of 0.2 mm global size were used with 6 layers of prism elements placed within the near-wall region of 1 mm or 20% diameter (whichever smaller) along the arterial wall boundaries. The mesh was optimised for the balance between computational accuracy and cost through mesh sensitivity test and the total number of mesh elements is in the order of 10^6^ eventually for all patient models.

#### Computational Methods

Our computational methods generally follow the previous studies [9, 20]. The 3D incompressible Navier-Stokes equations were solved to simulate the blood flow in the reconstructed anatomical models, using the commercial CFD package ANSYS CFX 19.2 (ANSYS, Inc. Canonsburg, USA). FFR was calculated under steady inflow condition to save computational cost. This follows our earlier investigation on the impact of flow pulsatility on CT-FFR computations, demonstrating 2.4% difference between CT-FFRs calculated under steady and pulsatile inflow conditions in return for eightfold acceleration: 2 h required for a steady inflow simulation and 16 h for a 5-cardiac-cycle pulsatile flow simulation [20]. In order to allow the system to reach a steady state after transient responses of Windkessel BCs, which will be described later in details, the simulations were carried out as transient simulation with a steady inflow condition and time step of 0.001 s. At each time step, the equation system was solved using algebraic multigrid solver until the root-mean-squared residual meets the convergence target 10^–5^. The flow was assumed to be laminar, and blood was modelled as homogenous and Newtonian fluid with its density and dynamic viscosity 1060 kg/m^3^ and 0.004 Pa s, respectively. The Newtonian assumption was adopted because coronary flow exhibits high shear rates (>10^3^ s^−1^), particularly near the vessel wall where pressure drops originate, placing blood rheology in the near-constant region of the non-Newtonian viscosity curve [21]. The vessel wall was approximated as rigid wall, where non-slip BCs were applied, and cardiac-induced wall motion was not incorporated. This is supported by the studies on the impact of wall motion on computed FFR, either via computational FSI (rigid wall FFR is 1.3% closer to invasive FFR than FSI [22]) or CFD with dynamic anatomical models from 4-dimensional CT (dynamic diameter variation induces FFR by 0.01 on average of 64 vessels [23]).

#### Inflow and Outflow Boundary Conditions

As introduced earlier, a steady inflow condition was applied at the aortic inlet. Since total cardiac output affects CT-FFR prediction for the same coronary tree model [24], it is vital to incorporate a patient-specific estimate of cardiac output. This could be done via 4-dimensional CT, i.e. stroke volume estimation based on end-diastolic–end-systolic difference of the ventricular cavity volume, or direct measurement using cardiac MRI or echocardiogram. At the same time, as our intention is to establish a workflow for CT-FFR computation without additional data acquisition modalities, an estimation of cardiac output using height and weight was adopted using the following equations [25].

1 CO=3.06×BSA+0.37,

2 $$BSA=\sqrt{\frac{H\times W}{3600}},$$

where CO is cardiac output (L/min), BSA is body surface area (m^2^), H and W are height (cm) and weight (kg), respectively.

To model the effect of peripheral vasculature, 2-element Windkessel model was used at each terminal branch of the 3D anatomical model (Fig. 1). The resistance component of a Windkessel was determined by a structured tree model proposed by Olufsen [26], which was shown to produce realistic resistance conditions for coronary flow simulations [27]. Briefly, the vascular network after the terminal boundary was modelled with a tree of asymmetric fractal-like bifurcations where each daughter branch of a bifurcation is further divided into asymmetric branches recursively. The branch had asymmetry ratio of 0.41, and the terminal branch diameter was set to 0.05 mm [26]. Note that a structured-tree-based resistance was used as a generic template in our model, and adjusted for adenosine-induced hyperaemia, based on several different approaches which are described later. The capacitance parameter was determined by setting the time constant (=1/RC) equal to 0.063 s following the literature [27]. Although the simulations were conducted with a steady inflow condition, the capacitance here plays an important role for the system to reach a steady state stably after the initial transient response to the Windkessel outflow BCs.

#### Models to Represent Hyperaemic Response

The computational model of CT-FFR requires a hyperaemic resistance. Here we propose a novel approach to derive a hyperaemic resistance individually for each patient, unlike conventional CT-FFR approaches assuming that the microvascular function can be represented by a population average, irrespectively to CAD patient or healthy individual.

For that, we introduce microvascular flow response (MFR), which is the hyperaemic flow rate increase in the microvasculature. Although CFR is more commonly used in this context, CFR represents vasodilatory capacity of microvasculature as well as the epicardial vessels. In the Windkessel BC, however, the resistance elements represent only the part of the coronary branch downstream to the 3D anatomical model (~microvasculature). MFR is therefore a more representative vasodilatory parameter to be used, also considering in vivo observations of epicardial coronary vessel dilation in response to adenosine [28]. In other words, if the Windkessel BC is tuned based on local CFR, the hyperaemic resistance could also reflect the vasodilatory response of the epicardial vessel hence overestimate the hyperaemic flow increase. With MFR, hyperaemic resistance can be defined using the following equation:

3 $${R}_{Hyp}=\frac{{R}_{Micro}}{MFR}$$

here ${R}_{Micro}$ is derived for each outlet of 3D model based on generic morphological approach as described earlier. MFR in our study is determined in five different approaches: (a) generic population average (conventional approach), (b) average of our PET patient cohort, (c) patient-specific based on CTP images ((c-i) per patient and (c-ii) per vascular territory) and (d) patient-specific based on our statistical MFR model (explained in details in the next section). In any of our models, a single MFR value was applied uniformly across the entire myocardium. This homogenous MFR assumption is based on our PET cohort data, showing a mean territorial (right coronary artery (RCA), left anterior descending (LAD) artery and left circumflex (LCx) artery regions) deviation of CFR as 0.13 ± 0.08 across the 101 patients, which is in line with clinical observation on non-obstructive CAD patients (maximum 7.5% territorial difference of microvascular resistance reserve [29].

Therefore, we apply our MFR-based BCs in 2 different ways, uniform for all branch outlets and branch-specific MFR based on spatially distributed CTP data sampled at each 3D anatomical model outlet. The former is an assumption in conventional CT-FFR including HeartFlow FFR_CT_ [4], and also used in most of our MFR models.

#### Patient-Specific MFR Model Based on Patient Demographic Metrics

The key aim of our computational model is to incorporate patient-specific response to the hyperaemia, as part of the outflow BCs, based on the data that is readily available for a patient that undergoes a CT coronary angiography. This is achieved via statistical model of hyperaemic response associated with typical demographic metrics such as, height, weight, age, sex, heart rate, blood pressure, and other medical conditions and health indicators. (Table 1). A univariate correlation analysis was performed first on each variable from the PET perfusion dataset against the MFR value (*n* = 101). This was followed by a multivariate correlation analysis between the MFR and the variables showing *P* < 0.10 in the univariate analysis. Then, a multiple regression was applied to build a model to predict MFR from the variables that were statistically significant in the multivariate correlation analysis. Statistical analysis was conducted using MATLAB (Mathworks, Natick, MA, USA). The final regression model for MFR, used to define outflow BCs in CFD, was evaluated for its statistical significance including prediction intervals computed for all combinations of the binary predictors. Note that, to be able to derive a representative MFR rather than CFR— the definitions are explained in the previous section—CFR of each patient was taken into the statistical model as the average of CFRs in the coronary territories (RCA, LAD and LCx) other than the territory of suspected stenosis. This however did not alter the overall CFR values significantly (MFR vs original CFR, 1.89 ± 0.48 vs 1.81 ± 0.47), as shown in Table 1.

#### Comparison and Validation

CT-FFR computation was performed for all 10 CTP cohort patients using anatomical models reconstructed from CT images, with various outflow BCs (different methods to determine MFR). These MFR models are: (a) conventional general population average, (b) patient population average based on PET cohort, (c-i) patient-specific and global average based on CTP, (c-ii) patient-specific and locally varied based on CTP, and (d) patient-specific global MFR based on our novel statistical model. For model (c-i) and (c-ii), local CFR values were sampled at each of the outlets of 3D anatomical model and used to calibrate the peripheral resistance in the BCs, following our earlier work [9]. All computations were conducted on a standard desktop workstation (Intel Xeon E5-2670 2.6 GHz, 128 GB RAM, 32 cores). Hereafter, these models will be called (a) *conventional MFR*, (b) *PET population average MFR*, (c-i) *CTP global MFR*, (c-ii) *CTP local MFR*, and (d) *demography-based MFR*. All the predicted FFR values for each patient were compared against the invasive FFR as the gold standard. Statistical comparisons here were made using a paired-samples Wilcoxon tests.

## Results

### Flow Response as a Function of Patient Metrics

The results of univariate and multivariate analyses, to investigate the potential association between MFR and demographic parameters, are summarised in Table 2. In the univariate analysis, sex, diabetes, smoking and hypertension turned out to be statistically significant (*P* < 0.05). In the multivariate analysis including all significant variables and age (*P* = 0.058) from the univariate analysis, sex, diabetes and smoking turned out as significantly associated with MFR (*P* = 0.039, 0.011 and 0.025, respectively).

**Table 2:** Regression coefficients and significance of various patient metrics on the overall MFR of a patient, derived from PET cohort (*n* = 101)

Parameters	Univariate		Multivariate		
	Coefficient	*P*-value	Coefficient	*P*-value	Standard error
Age	− 0.0098	0.0583	− 0.0017	0.7371	–
Sex	**0.2302**	**0.0231**	**0.2015**	**0.0386**	**0.0934**
Pain limitation	− 0.1440	0.1401	–	–	–
Breathlessness	− 0.1237	0.2051	–	–	–
Diabetes	**− 0.3334**	**0.0040**	**− 0.2859**	**0.0106**	**0.1082**
Smoking	**0.2917**	**0.0097**	**0.2474**	**0.0246**	**0.1043**
Hypertension	**− 0.1949**	**0.0461**	− 0.1251	0.1747	–
Hyperlipidaemia	− 0.0753	0.4488	–	–	–
Family history	0.0651	0.5063	–	–	–
Rest HR	− 0.0061	0.1090	–	–	–
Rest systolic BP	− 0.0037	0.0953	–	–	–
Rest diastolic BP	− 0.0019	0.6293	–	–	–
Stress HR	0.0010	0.7459	–	–	–
Stress systolic BP	− 0.0036	0.1362	–	–	–
Stress diastolic BP	− 0.0046	0.2688	–	–	–

Variables with *P* < 0.10 in the univariate analysis (highlighted by bold) were taken into the multivariate analysis. The variables highlighted by bold letter for multivariate analysis are those with *P* < 0.05

Based only on the statistically significant contributing factors to MFR in the multivariate analysis, a multivariate regression model was derived. The formula for MFR is:

4 MFR=1.95+0.22Sx-0.30Db+0.28Sm

here, Sx is the sex of the patient (1 if male) Db is diabetes status (1 if diabetes), and. Sm is the patient’s smoking status (1 if smoker). Note that smoker status is self-declared. The model evaluation in Table 3 shows that it has overall statistical significance, with adjusted *R*^2^ 0.16 and predictive uncertainty 2.31.

**Table 3:** Evaluation of the regression model for MFR (Eq. 4)

	Adjusted *R*^2^	Significance	Predictive uncertainty
Overall model	0.16	*P* < 0.001	2.31

Variables	Coefficient	Standard error	Confidence interval
Sex	0.22	0.093	(0.036, 0.407)
Diabetes	− 0.30	0.107	(− 0.524, − 0.096)
Smoking	0.28	0.104	(0.057, 0.473)

The overall model significance is the result of F-test on the regression model, and the predictive uncertainty is the mean 95% prediction-interval width across all predictor combinations

### Predicted vs. Measured MFR

The MFR values used in each CFD simulation of CTP cohort patient, including those in other 3 approaches, is summarised in Table 4. The measured MFR values (2.14 ± 0.51 for PET and 2.32 ± 0.57 for the CTP cohorts, respectively) are lower than the conventional assumption of 3.5. Diseased branch CFRs are lower than the global value (1.72 ± 0.67 and 2.32 ± 0.57). Our demography-based estimate captured the MFR of CTP cohort on average (2.34 ± 0.55 vs 2.32 ± 0.57), although there are discrepancies in some patients, e.g. Patient 7’s measured global MFR is 3.31 that is predicted as 2.45 by our model.

**Table 4:** MFR values used in CT-FFR simulations of CTP cohort

Patient	Conventional (a) [30]	PET population average (b)	Patient-specific based on CTP		Demography-based estimate (d)
			Global (c-i)	Diseased branch CFR (c-ii)	
1	3.50	2.14 ± 0.51	1.28	0.82	1.87
2			1.83	1.25	2.17
3			2.32	1.71	2.45
4			2.61	2.01	2.45
5			2.49	1.23	2.45
6			2.31	2.10	2.45
7			3.31	3.13	2.45
8			2.83	2.31	2.13
9			2.03	1.95	2.45
10			2.46	1.07	2.45
				1.29	
Mean ± SD			2.32 ± 0.57	1.72 ± 0.67	2.34 ± 0.20

The patient population average is for our PET patient cohort. For the diseased branch of CTP cohort, CFR is shown as MFR does not include diseased branch (see the definition in Sect. 2.2.4). Demography-based estimates are from Eq. 4

### Predicted vs. Invasive FFR

**Fig. 2:**
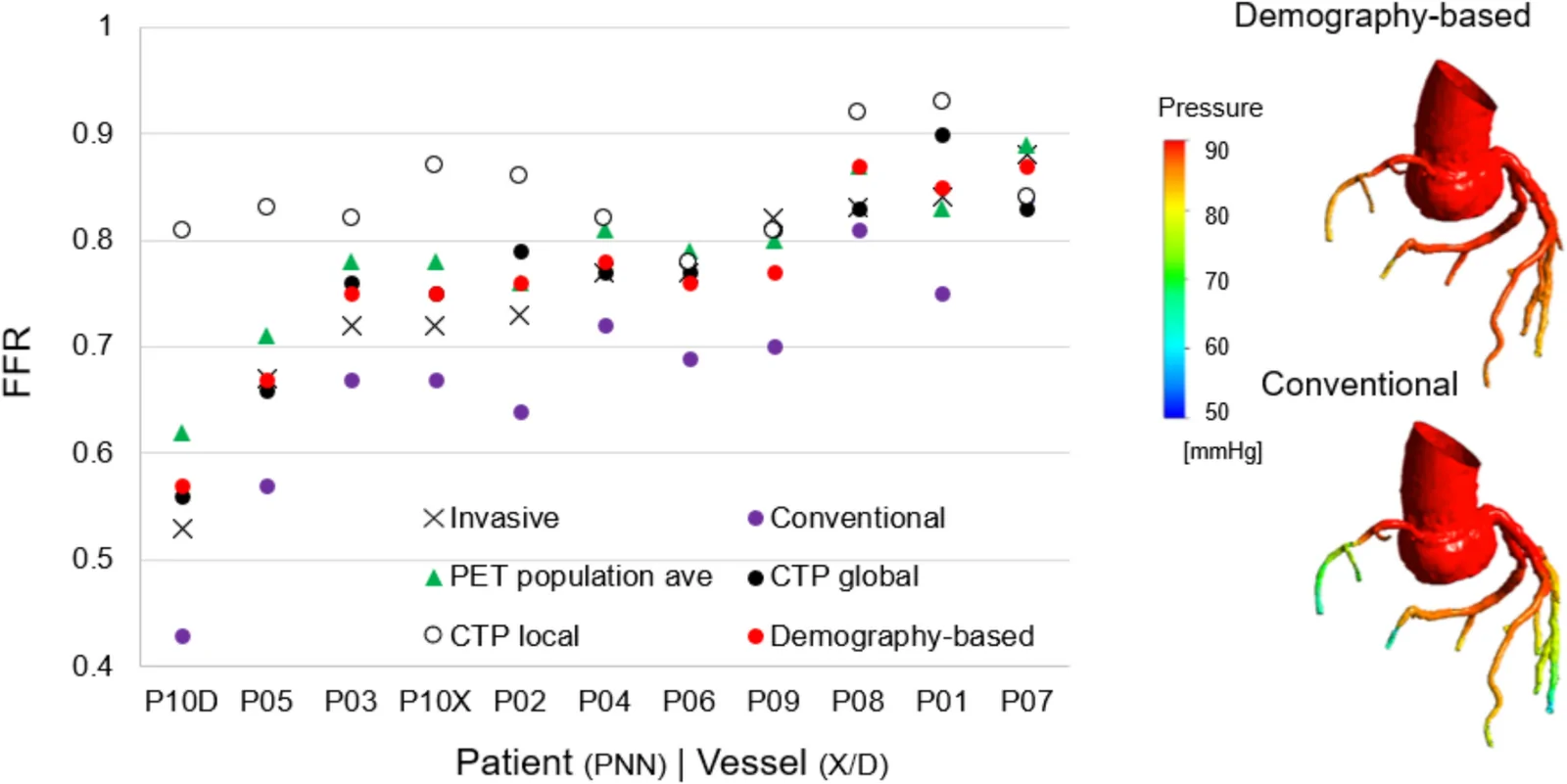
Predicted FFRs as the results of CFD*.* Comparison of predicted FFRs using various approaches against invasive FFR (left), and pressure distribution along the coronary tree in two representative cases (right, Patient 7 with statistical patient-specific MFR and conventional MFR model

**Fig. 3:**
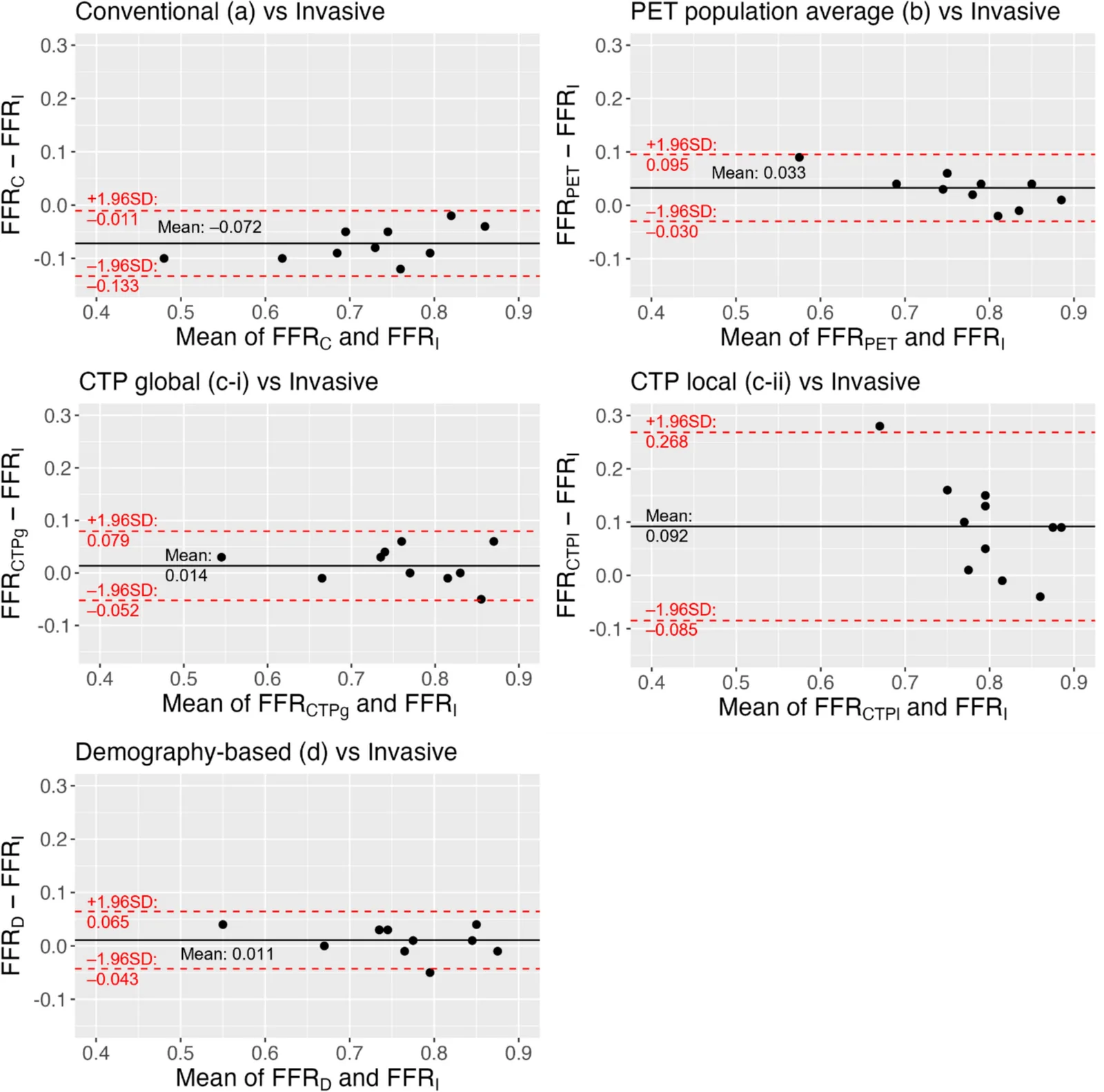
Bland-Altman plot to compare computed FFRs with different outflow conditions against the invasive FFRs. Note that $FF{R}_{I}$, $FF{R}_{C}$, $FF{R}_{PET}$, $FF{R}_{CTPg}$, $FF{R}_{CTPl}$ and $FF{R}_{D}$ are FFRs from invasive, conventional, PET population average, CTP global, CTP local and demography-based approaches

The FFR values derived from CFD are presented in Fig. 2 along with pressure distribution of representative cases (Patient 7 with our outflow BC model and conventional one). The predicted FFR values are also compared against the invasive FFRs in Bland-Altman plot in Fig. 3. The quantitative FFR results—both predicted and invasive—and for all the CTP cohort patients are summarised in Table 5.

**Table 5:** FFR values obtained by various hyperaemic flow response models applied to the downstream outlet boundary condition

Patient	Lesion	Invasive FFR	CT-FFR values with various MFR models				
			Conventional (a)	PET population average (b)	CTP global (c-i)	CTP local (c-ii)	Demography-based (d)
1	LCx	0.84	**0.75**	0.83	0.90	0.93	0.85
2	LAD	**0.73**	**0.64**	**0.76**	**0.79**	0.86	**0.76**
3	LAD	**0.72**	**0.67**	**0.78**	**0.76**	0.82	**0.75**
4	LAD	**0.77**	**0.72**	0.81	**0.77**	0.82	**0.78**
5	RCA	**0.67**	**0.57**	**0.71**	**0.66**	0.83	**0.67**
6	LAD	**0.77**	**0.69**	**0.79**	**0.77**	**0.78**	**0.76**
7	LCx	0.88	0.84	0.89	0.83	0.84	0.87
8	LAD	0.83	0.81	0.87	0.83	0.92	0.87
9	LAD	0.82	0.70	**0.80**	0.81	0.81	**0.77**
10	LAD	**0.53**	**0.43**	**0.62**	**0.56**	0.81	**0.57**
	LCx	**0.72**	**0.67**	**0.78**	**0.75**	0.87	**0.75**
Mean ± SD		0.75 ± 0.10	0.68 ± 0.11	0.79 ± 0.07	0.74 ± 0.10	0.84 ± 0.05	0.76 ± 0.09
Mean difference vs invasive FFR		–	− 0.073	0.038	− 0.019	0.092	0.011
*P* value vs. invasive FFR		–	0.004	0.013	0.233	0.011	0.217

Invasive FFRs of those patients are also shown, and values indicating positive diagnostic results (FFR < 0.8) are highlighted in bold

There is a wide variety of disease levels present in this cohort, in terms of the invasive FFR values (0.53–0.88). For five out of the eleven vessels analysed, FFR was predicted between 0.75 and 0.85, close to the FFR = 0.8 cut-off. Amongst those, FFR for Patients 4 and 6 was 0.77, which resides in the commonly denoted “grey zone” of FFR (FFR = 0.75–0.80). There are two patients in the more severe category, with FFR of 0.67 and 0.53, respectively. Patient 10 has multivessel disease, with both LAD and LCx containing functionally significant stenoses that has FFR < 0.8.

Overall, the predicted FFRs by all models follow the trend of invasive FFR (Fig. 2 and Table 5). However, the FFRs derived by the conventional model are lower than most of the other models’ prediction and invasive FFR. The trend is also indicated in the Bland-Altman plot (Fig. 3) by the negative bias of − 0.072. The figure also shows a relatively narrow range of agreement, except for the CTP local MFR model. Quantitative comparisons of FFR values indicate a significant discrepancy against invasive measurement (0.75 ± 0.10) for conventional (0.68 ± 0.11, *P* = 0.004), PET population average (0.79 ± 0.07, *P* = 0.013) and CTP local (0.84 ± 0.05, *P* = 0.011). On the other hand, the CTP global MFR and demography-based models predicted FFR values (0.74 ± 0.10 and 0.76 ± 0.09, respectively) more closely agreeing with the invasive FFRs (*P* = 0.233 and *P* = 0.217, respectively), although the predictions of those models tended to be higher (Figs. 2 and 3).

### Potential Impact on Diagnostic Accuracy

Table 6 shows the sensitivity, specificity, and accuracy of the FFRs predicted using various methods. Balanced accuracy is also included as the patient dataset was skewed towards more positive cases than negative. Here, as mentioned earlier, a conventional threshold of 0.80 is used for positive diagnostic outcome.

**Table 6:** Sensitivity, specificity, positive predictive value (PPV), negative predictive value (NPV), accuracy (Acc) and balanced accuracy (BAcc) for various CT-FFR methods compared to the classification based on the invasive FFR (with FFR = 0.8 as the threshold)

FFR computation method	Sensitivity (%)	Specificity (%)	PPV (%)	NPV (%)	Acc (%)	BAcc (%)
Conventional MFR	100	50	78	100	82	89
PET population average MFR	86	100	100	80	91	90
CTP global MFR	100	100	100	100	100	100
Demography-based MFR	100	75	88	100	91	94

With all models except for the population average MFR, sensitivity of the prediction is all high as 100% whereas specificity varies between 50 and 100%. Accuracy of the prediction is 100% with the MRF model fully tuned by perfusion (CTP) data. Accuracy of the conventional model is lower at 82%, and the other two models showed in 91% accuracy, which is not as high as the fully tuned model but markedly higher than the conventional model.

## Discussion

In this study, we proposed a novel statistical model to estimate the hyperaemic response of myocardium, associated with routinely recorded demographic variables, to be used as part of patient-specific outflow condition of CT-based FFR computations. To define outflow condition in CT-FFR computations is one of the areas that have not been explored thoroughly and our study was aimed to provide an additional insight in this area and propose a novel methodological framework. The results showed that the predicted FFRs using our model agree well with the invasively measured FFR in 10 ‘unseen’ patients in a cohort substantially different (i.e. from a different hospital) from the one for which the model was calibrated. Our model, without needing any additional data to CTCA, predicated FFR values better agreeing with invasive measurement than other approaches including conventional CT-FFR model. In the following sections, we discuss validity and interpretation of the model results, and limitations in details.

### Association Between MFR and Demographic Parameters

Our multivariate analysis showed that sex, diabetes and smoking are the key contributing factors to predict MFR. Amongst these, with the regression coefficient of 0.22, male has been shown to have a higher tendency for high myocardial hyperaemic response. Female sex has been shown to have a higher prevalence of coronary microvascular dysfunction (CMD) [31], and exhibiting cardiovascular disease symptoms have a much higher probability of having microvascular dysfunction relative to male patients [32]. These might explain the trend in our data, although the lack of other evidence of CMD in our PET cohort does not offer a conclusive explanation.

Diabetes turned out to be the most significant factor that affects MFR with its regression coefficient − 0.30. It is well documented that microvascular dilatory capability and CFR are lowered in diabetic patients, due to increased formation of plaques in both macrovascular and microvascular sections of the coronary circulatory system, caused by hyperglycaemia, sustained high levels of blood sugar causing damage to arterial linings [33, 34].

Smoking is also one factor associated with CFR (regression coefficient 0.265), indicating that the smokers in our PET cohort tend to have more responsive myocardium to hyperaemia. This is counterintuitive and contradicting to the literature, e.g. studies on CFR shows that smoking causes damage to microvascular circulatory function [35], even at a mild level [36]. There is however one possible explanation is the well-known “smoker’s paradox” which is essentially a sampling bias [37]: smokers tend to present in clinics at less severe stages of CAD (6.1% of non-smokers vs. 3.4% smokers had severe disease (*P* < 0.0001) in ACUITY Trial at baseline [38]) although they might have symptoms common amongst more severe stages of CAD such as chest pain or breathlessness associated with lungs. Additionally, some reports smokers present at a younger age than non-smokers (e.g. mean age 58.2 vs. 71.7, *P* < 0.001 in [39]). These patients could exhibit more responsive microvasculature (i.e. higher CFR) than non-smokers through cardiac function tests (e.g. PET), which does not mean smoking improves CFR.

Our results did not show a significant association between MFR and other demographic parameters such as age. However, association of CFR to age, hypertension, hyperlipidaemia and family history has been reported [40, 41]. This may be explained by the characteristics of our patient cohort (PET cohort in Table 1), which has high and narrow age range (70 ± 9 years) and includes only patients with symptoms. Indeed, a similar regression-based model for myocardial blood flow, derived by Knot et al. based on a larger cohort (*n* = 242) with lower mean age (56.9 years) [19], involves age and LV mass in the prediction. Our current model, Eq. 4, allows to produce 8 different MFR values in the range between 1.65 and 2.45, by taking 3 binary inputs (sex, smoking, and diabetes). The results of CT-FFR calculation demonstrated that this variation effectively facilitates an improved prediction of FFR, but inclusion of continuous input parameters such as age would make the model more versatile.

### Quantitative Myocardial Flow Response Estimations

In an idealised healthy individual, CFR is reported to be approximately between 3.5 and 4.0 and CFR value less than 2.0 is considered as a defect in myocardial flow response [42]. Both of our patient datasets—PET and CTP—show a flow response lower than 3.5 (Table 3). Although CFR (MFR) of the two datasets were calculated in different ways, both CFR ranges are significantly lower than the healthy population average assumption of 3.5. PET-based CFR (1.89 ± 0.48) was a routine clinical measurement by experienced radiographers using Siemens Syngo system, whereas CTP-based CFR (2.36 ± 0.52) was calculated directly from the perfusion image using an in-house MATLAB programme. The discrepancy between the CTP dataset and the PET dataset is likely due to sex, the CTP dataset has only male patients. Our statistical model captured the difference and MFR predicted by our model for the CTP patients is 2.34 ± 0.55 vs. the measured value 2.32 ± 0.57.

In our modelling framework, global impact of microvascular diseases is considered as an influential factor to MFR. The potential impact of local ischaemic disease such as myocardial infarction is not considered, assuming that the CT-FFR would primarily be used for patients in earlier stage of the disease. It is therefore unlikely for a single branch of an artery to have significant microvascular defect, other than local ischaemia, and healthy microvascular vasodilatory function in other branches [43]. In our data in Table 4, the CFR of diseased branch is significantly reduced in comparison to the global mean. More specifically for Patient 10 with two diseased vessels, one moderate and one severe disease based on invasive FFR (Table 5), has a relatively healthy flow response overall of 2.46 (Table 4). The branch-wise CFRs are however markedly lower (1.07 and 1.29) suggesting that the low CFR values could indeed by significantly affected by the epicardial stenosis, also seen in Patient 5.

### Quantitative Accuracy of the CT-FFR Calculations

Comparison of predicted FFRs against the invasive measurement (Table 4) showed that the conventional FFR algorithm assuming MFR of general population average underestimates FFR (i.e. overestimates the functional disease severity). This is expected, as also shown in our earlier study [9]. CT-FFR computation with our demography-based MFR prediction model resulted the closest match with invasive FFR, followed by the CTP-based patient-specific global flow response by small margin (FFR discrepancy of 0.011 and 0.019 from invasive, respectively). In the present (CTP) patient cohort, the largest MFR discrepancy in Patient 7 between different MFR calculation approaches was not reflected as a large variability of FFR. That is likely to be because of less severe stenosis, represented by the invasive FFR = 0.88, which is less sensitive to the outflow condition [9, 24]. The impact of unsuccessful MFR prediction by the demography-based model warrants a detailed investigation in the future studies with larger patient cohort. The locally calibrated MFR by CTP data shows significant discrepancies from the invasive measurement, overestimating the FFR with the error of 0.092 (Table 5). Intuitively, local resistance calibration would improve the accuracy of model. It should however be noted that the CTP-based local CFR for diseased branches are substantially lower than the global values, i.e. average of other branches (Table 4), because it includes the impact of both microvascular and epicardial vessel responses, hence the use of local CFR resulted in a lower flow in the diseased branch, a smaller pressure drop across the lesion and higher FFR. This is clearly demonstrated in the Bland–Altman plot (Fig. 3) as a large positive bias. Our earlier study [9] highlighted the impact of outflow BC of CT-FFR computation but the new results provide an additional insight that the BC should be tuned to what represents individual physiology well. These results support the use of MFR as a patient-specific but global parameter rather than local, also in line with the literature indicating that epicardial stenosis should not affect microvascular resistance [43].

The average quantitative change of FFR by introduction of our model is 0.08, from 0.68 to 0.76, from conventional to our model. Whilst this may look small, it is fourfold larger than the test-retest variability 0.019 of invasive FFR of [44, 45]. Although a larger cohort study would be needed to confirm its efficacy more strongly, this comparison suggests that the proposed demography-based model has a significant practical value, since it produces more reliable FFR values than conventional approach, without the need of additional imaging data.

### Potential Impact on Clinical Diagnostic Classification

Computational FFR prediction with our demography-based outflow BC resulted in a high sensitivity and diagnostic accuracy (100% and 94%) in comparison to the classification based on invasive FFR, which is superior to the conventional approach (balanced accuracy 89%). In consistent with clinical studies [46, 47] using conventional CT-FFR algorithms, our analyses using the conventional scheme show a high sensitivity and negative predictive value, i.e. positive cases are almost always detected, but low specificity and positive predictive value, 2 out of 4 ‘healthy’ cases are misclassified (Table 5). On the contrary, as expected from the over-estimated FFR values, the locally calibrated MFR model classified 9 out of the 10 patients as non-functionally severe state, substantially different from invasive-FFR-based classifications. At the same time, when used as a globally uniform MFR, the CPT-based calibration improved the classification and resulted in 100% accuracy.

The proposed CT-FFR workflow with demography-based MFR model is a high compatibility with the current clinical pathway, since it only requires CTCA and commonly available demographic parameters. Whilst non-invasive functional testing such as PET, SPECT, perfusion MRI, and more recently CT perfusion may be preferred as the direct measure of myocardial ischaemia [48], perfusion-based imaging is not commonly conducted due to lack of facility, and/or cost both in monetary and skilled operator terms. Except for MRI perfusion, the other perfusion imaging techniques also incur a radiation dose on the patient. Moreover, since CTCA is recommended as the first line of investigation for CAD especially in the USA and UK [13], it makes sense to utilise CT-FFR for the subset of patients left after morphologically severe lesions that can be diagnosed reliably in CTCA [13]. Our approach is to improve the CT-FFR computation without any extra data collection, hence, to enhance the known benefits of CT-FFR such as non-invasive diagnosis of coronary heart disease and reduction of catheter-based procedures [1–4]. Furthermore, this approach could easily be integrated in the training process of machine-learning-based models that are eagerly explored in these years [49].

Since FFR is primarily a binary diagnostic tool, it is prone to a diagnostic accuracy concern especially for patients around the threshold (e.g. in grey zone). Although diagnosis of those will not be blindly adhered to, and additional testing or conditions would be considered [50], errors in calculation can produce a vastly different diagnostic result and therefore treatment decision. To support the clinical diagnostics further, our model could incorporate statistical confidence with the predicted FFRs associated with the uncertainty in the demographic model, similarly to our earlier attempt to present a range of FFR rather than single value [9]. Thus, the likelihood of a patient falling into a certain classification can be provide to the clinicians who could diagnose the patient in a more holistic manner.

### Limitations and Future Directions

Our study was conducted with a relatively low number of patients (*n* = 10) with skewed demographic (all male) for our validation against invasive FFR. Based on the sensitivity study as part of our previous work [9], the gender-specific − 0.22 difference of MFR in Eq. 4 could elevate FFR by 0.022 at the maximum. Although data of patients having both CTCA and invasive FFR are not routinely available, validation of the method in a larger and a more balanced cohort is essential to consolidate our finding. It should also be noted that the conventional CT-FFR technique used in this study was of its simplest form based on the literature [1], and not meant to be a reproduction of commercially available methodologies/services that may have more sophisticated additional algorithms.

In our demography-based MFR model, many of the input variables are binary such as diabetes and smoker status. Those labels do not further describe for example how severe the diabetes is, duration and degree of the smoking. A more detailed record of these parameters would offer further fine tuning of the MFR model, along with refinement of the model formulation such as inclusion of interactions. Additionally, it is important to expand the “training” PET dataset including a wider variety of patients, especially age range and inclusion of healthy/healthier subjects, as discussed earlier. Inclusion of normal patients’ data, although cardiovascular MR would be more suitable [51] for those than PET, would also make the model more widely applicable.

We also acknowledge that our computational methods are simplified by assuming Newtonian blood viscosity, rigid wall and steady inflow conditions although we consider those assumptions have limited impact on FFR. Whilst the limitations above altogether would not de-value the potential of the proposed algorithm especially in this proof-of-concept stage, a more thorough comparative study of CT-FFR variants is warranted in the future.

### Summary

A novel computational CT-FFR framework with an improved patient-specific outflow boundary conditions based on the demographic parameters was proposed. The boundary condition is based on a PET perfusion dataset to derive a multivariate regression model associating hyperaemic flow response and patient metrics that are available in routine clinical record. This facilitated the novel framework to derive improved FFR predictions without needing any additional imaging data other than CTCA, which is used in the CT-FFR algorithms currently available. The CT-FFR computations with the novel outflow boundary conditions, based on patient’s sex, diabetes and smoking status, predicted FFR values agreeing well with the invasively measured FFR. Whilst the agreement of FFR between the proposed model and invasive FFR was second best after that of CT-FFR fully calibrated with CT-perfusion-based outflow conditions, it was superior to the conventional CT-FFR model without requirement of any additional imaging data (diagnostic accuracy vs invasive FFR: 100, 91 and 82% for CT-FFR with CTP-based MFR, demography-based MFR, and conventional, respectively). The study therefore demonstrated, although in a proof-of-concept stage, the efficacy and practicality of the proposed outflow BC model that could improve the existing CT-FFR framework without any additional data outside standard clinical routine.

## Data Availability

The datasets for this article are not publicly available due to concerns regarding participant/patient anonymity. Requests to access the datasets should be directed to the corresponding author.
